# The diagnostic potential of urinary volatile organic compounds for colorectal neoplasia in Lynch syndrome—A prospective longitudinal study

**DOI:** 10.1002/ijc.70140

**Published:** 2025-09-23

**Authors:** Elsa L. S. A. van Liere, Dewkoemar Ramsoekh, Trenton K. Stewart, Emma Daulton, Maarten A. J. M. Jacobs, Evelien Dekker, Sofie Bosch, James A. Covington, Tim G. J. de Meij, Nanne K. H. de Boer

**Affiliations:** ^1^ Department of Gastroenterology and Hepatology Amsterdam University Medical Center Amsterdam The Netherlands; ^2^ Amsterdam Gastroenterology Endocrinology Metabolism Research Institute Amsterdam The Netherlands; ^3^ School of Engineering University of Warwick Coventry UK; ^4^ Department of Paediatric Gastroenterology Amsterdam University Medical Center Amsterdam The Netherlands

**Keywords:** biomarkers, colorectal cancer, Lynch syndrome, surveillance, volatile organic compounds

## Abstract

Post‐colonoscopy colorectal cancer (CRC) rates and colonoscopy burden are considerable in Lynch syndrome. Urinary volatile organic compounds (VOCs) have shown promise as a patient‐friendly alternative to faecal biomarkers for colorectal neoplasia detection. To evaluate the potential of urinary VOCs to guide optimal colonoscopy intervals in Lynch syndrome, we performed an exploratory prospective longitudinal study in urine collected by individuals with Lynch syndrome before and after colonoscopy. VOC patterns were analysed by field asymmetric ion mobility spectrometry (FAIMS) and gas chromatography‐ion mobility spectrometry (GC‐IMS) followed by machine learning algorithms. Gas chromatography time‐of‐flight mass spectrometry analysed the abundance of individual VOCs. Among 98 included individuals (median 51y, 58% female), 34 had relevant neoplasia at colonoscopy, including 28 non‐advanced adenomas, 3 advanced adenomas, 2 CRCs, and 1 advanced serrated lesion. For GC‐IMS, the respective sensitivity and negative predictive value for relevant neoplasia were 65% and 79% (70% specificity); for FAIMS, 74% and 75% (42% specificity). VOC patterns differed before and after polypectomy (AUC 0.84), while after polypectomy they resembled those of individuals without neoplasia (AUC 0.52). Non‐advanced adenoma presence was associated with increased urinary abundance of decanoic acid (fatty acid). Diagnostic accuracy of urinary VOC patterns for relevant neoplasia was influenced by sample size and external confounders and was lower than for faecal VOC patterns among 50 individuals who had also collected faeces. Urinary VOCs hold promise as non‐invasive biomarkers for postponing colonoscopy and for follow‐up after polypectomy in Lynch syndrome, though large validation studies are needed that also assess stability and accuracy compared to faecal VOCs.

## Graphical abstract

What's new?

Lynch syndrome demands lifelong colonoscopy surveillance to early detect and treat colorectal cancer (CRC) and prevent CRC by removing adenomas. However, colonoscopy overuse and post‐colonoscopy CRC rates among individuals with Lynch syndrome are problematic. This study explored the use of urinary volatile organic compounds (VOCs) as biomarkers to help overcome colonoscopy challenges for Lynch syndrome. VOCs showed 65%–74% sensitivity and 42%–70% specificity for CRC and adenomas. VOC patterns differed before and after polypectomy, while VOCs post‐polypectomy resembled those of individuals without neoplasia. Thus, urinary VOCs are a promising patient‐friendly alternative to faecal biomarkers for guiding optimal colonoscopy intervals in Lynch syndrome.

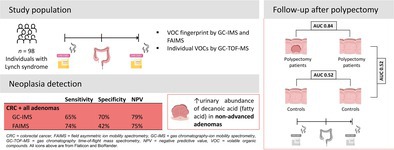

**Abbreviations**
AUC: area under the curve
CRC: colorectal cancer
FAIMS: field asymmetric ion mobility spectrometry
GC‐IMS: gas chromatography‐ion mobility spectrometry
GC‐TOF‐MS: gas chromatography time‐of‐flight mass spectrometry
IQR: interquartile range
NPV: negative predictive value
VOCs: volatile organic compounds

## 1 INTRODUCTION

An estimated 1 in 279 individuals has Lynch syndrome, which is associated with an increased lifetime risk for colorectal cancer (CRC) of 15%–70% depending on pathogenic gene variant.^1^, ^2^ CRC incidence and mortality have reduced significantly in this population, due to early detection of CRC and removal of adenomas through 2‐yearly colonoscopies from age 25 or 35 years.^2^, ^3^

Nevertheless, this regular and lifelong colonoscopy surveillance programme is resource‐intensive and costly, and experienced by individuals as burdensome, time‐consuming, and detrimental to quality of life—leading to suboptimal adherence.^4^, ^5^, ^6^, ^7^, ^8^ It would be of value to reduce the number of unnecessary colonoscopies (those negative for relevant neoplasia), which may account for approximately 70% of colonoscopies performed for Lynch syndrome.^9^, ^10^ On the contrary, despite strict surveillance, post‐colonoscopy CRCs still occur, partly due to missed or incompletely resected adenomas.^11^, ^12^ As such, some individuals may benefit from a shorter colonoscopy interval.

Colonoscopy overuse and post‐colonoscopy CRC rates in Lynch syndrome may be reduced by non‐invasive biomarkers guiding optimal colonoscopy intervals.^13^ Faecal biomarkers would be the most obvious candidates, as faeces directly reflect the intestinal state, and they have recently shown promising results in Lynch syndrome.^13^, ^14^, ^15^ Nevertheless, challenges of faecal sampling may be patient acceptability, external confounders related to microbiome composition (e.g., diet, smoking, medication), and that it cannot be obtained on demand.^15^, ^16^, ^17^, ^18^, ^19^, ^20^ These challenges may be overcome by the use of urinary biomarkers.^21^, ^22^, ^23^, ^24^ Promising urinary biomarkers for CRC detection are volatile organic compounds (VOCs), yet their potential for adenoma detection and for follow‐up after polypectomy remains to be determined.^25^, ^26^ Urinary VOCs have also not been studied in the context of Lynch syndrome.

Therefore, we designed a prospective longitudinal study in individuals with Lynch syndrome to assess the performance of urinary VOCs for detection of CRC and adenomas and for intra‐individual follow‐up after polypectomy. A non‐invasive patient‐friendly biomarker panel of urinary VOCs may allow more effective and personalised surveillance in Lynch syndrome.

## 2 MATERIALS AND METHODS

### 2.1 Study participants and sample collection

This prospective longitudinal single‐centre study was performed between November 2020 and February 2023, as part of a larger study on diagnostic biomarkers for colorectal neoplasia in Lynch syndrome.^14^, ^15^ For the current study, we consecutively included individuals with a pathogenic germline mismatch repair gene variant and without previous (sub‐)total colectomy, who were scheduled for surveillance colonoscopy at an academic hospital in the Netherlands (Amsterdam UMC). Individuals participating in our study collected urine in plastic containers (30 mL, Thermo Scientific, USA) within 3 months before colonoscopy and bowel preparation, as well as within 3–6 months after colonoscopy. At the time of sample collection, participants also completed an online questionnaire on various external factors possibly influencing the accuracy of urinary VOCs. Participants were instructed to collect midstream spot urine after overnight fasting and to avoid collection during menstruation. Samples were stored in the participant's own freezer within 1 h following collection and transported to the hospital either by the participant using icepacks or by a researcher using dry ice. On arrival at the hospital, samples were stored at −80°C until further analysis.

### 2.2 Colonoscopy and histopathology

Colonoscopy and histopathology workflows are detailed in our previous publications,^14^, ^15^ and followed the European Society of Gastrointestinal Endoscopy recommendations.^2^, ^27^ In short, colonoscopies were performed by experienced endoscopists using high‐definition white light endoscopy and, where appropriate, advanced imaging techniques. The cecum was intubated, and the Boston Bowel Preparation Score was ≥2 in each segment. Except for hyperplastic polyps ≤5 mm in the sigmoid or rectum,^28^ all detected neoplasia were resected using standard techniques and evaluated by experienced gastrointestinal pathologists according to the Vienna classification.^29^ Data on neoplasia size and location was retrieved from the endoscopy report. Neoplasia in the splenic flexure, descending colon, sigmoid or rectum were classified as distally located.

### 2.3 Sample and data analysis

After samples were defrosted in a fridge overnight, 5 mL of manually homogenised urine was aliquoted into glass containers (20 mL headspace vials with crimp cap, Thames Restek, UK), which were transported on dry ice to the University of Warwick (UK) for analysis. Researchers blinded to colonoscopy outcomes analysed headspace VOCs of urine samples by three advanced systems, which analyse different chemical windows and have different (dis)advantages: gas chromatography time‐of‐flight mass spectrometry (GC‐TOF‐MS), gas chromatography‐ion mobility spectrometry (GC‐IMS) and field asymmetric ion mobility spectrometry (FAIMS). GC‐TOF‐MS analyses were performed in one batch after a median storage period of 7 months (interquartile range [IQR] 5–9). GC‐IMS and FAIMS analyses were both performed in three batches to ensure that samples were analysed within 12 months (median storage period was 7 months [IQR 5–10]), as urinary VOCs are considered stable for this period when stored frozen.^30^ Of note, samples before and after colonoscopy from the same individual were analysed within the same batch. The analytical methodologies of GC‐TOF‐MS, GC‐IMS and FAIMS are detailed in Appendix S1.

VOC data analysis was performed according to the protocol used in our previous study.^15^ In short, the GC‐IMS and FAIMS output was pre‐processed, and then urinary VOC patterns (the “VOC‐fingerprint”) were compared between groups detailed below, using a custom platform in R that first performs binary class prediction by 10‐fold cross validation and then tests different machine learning algorithms (XGBoost, support vector machine, Gaussian process, random forest and sparse logistic regression). The feature location plots were reviewed to confirm that discriminatory VOCs were within the chemical output. The GC‐TOF‐MS output was also pre‐processed, and then peaks were matched to individual VOCs registered in the NIST 2020 library, using forward and reverse searching. Patient characteristics were compared between groups using the Mann–Whitney *U* test for continuous variables and the Chi‐square or Fisher's exact test for categorical variables.

### 2.4 Outcome parameters

Classification of individuals was based on the most relevant neoplasia at colonoscopy, which included CRC, advanced adenoma (≥10 mm, high‐grade dysplasia or villous component^31^), advanced serrated lesion (≥10 mm or dysplasia^32^) and non‐advanced adenoma. Those with non‐advanced serrated lesion only, or without colorectal lesions, were considered as controls.^33^

**FIGURE 1:**
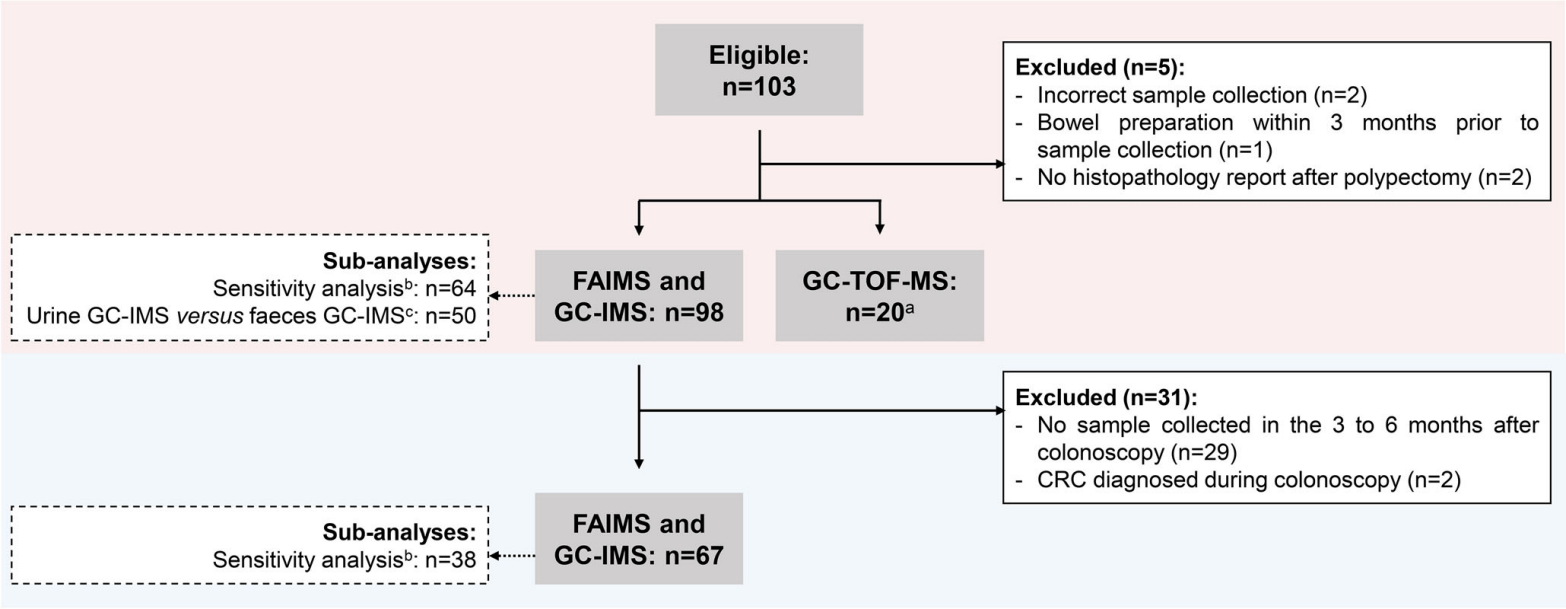
Flow diagram showing the different analyses conducted, including numbers of patients analysed, to evaluate the performance of urinary volatile organic compounds for detection of colorectal neoplasia (upper red box) and for intra‐individual follow‐up after polypectomy (lower blue box) in Lynch syndrome. Abbrevations: CRC, colorectal cancer; FAIMS, field asymmetric ion mobility spectrometry; GC‐IMS, gas chromatography‐ion mobility spectrometry; GC‐TOF‐MS, gas chromatography time‐of‐flight mass spectrometry. ^a^GC‐TOF‐MS was performed in a random selection of 10 non‐advanced adenomas and 10 controls. ^b^For sensitivity analysis, individuals with and without relevant colorectal neoplasia were matched 1:1 on possible confounders: gender, age, smoking habits and dietary habits. Only *n* = 2 non‐advanced adenomas could not be matched and therefore were excluded. ^c^The sub‐analysis urine FAIMS versus faeces FAIMS was not feasible, due to insufficient sample numbers and different FAIMS methodology for urine and faecal samples.

An overview of the outcome parameters is provided in Figure 1. Using samples collected before colonoscopy, we assessed the performance of urinary VOC patterns (the “VOC‐fingerprint”, analysed using GC‐IMS and FAIMS) for the detection of relevant colorectal neoplasia. To assess the performance of urinary VOC patterns for intra‐individual follow‐up after polypectomy, we compared samples collected before and after colonoscopy of both controls and individuals with complete polypectomy. To evaluate whether external factors influenced the performance of urinary VOC patterns for neoplasia detection and follow‐up, we performed sensitivity analyses in individuals with and without neoplasia matched 1:1 on gender, age (±10 years), dietary habits (yes/no vegetarian) and smoking habits (yes/no active smoker).^18^, ^19^, ^20^ Next, we compared the diagnostic performance for relevant neoplasia between urinary and faecal VOC patterns, analysed by identical GC‐IMS methodology, for which we used faecal samples that were collected in our previous study^15^ within 30 days of the urine sample. This comparison was not feasible for FAIMS, due to insufficient sample numbers and different FAIMS methodology for urine and faecal samples.

GC‐TOF‐MS analyses were performed in a random subset of controls and individuals with non‐advanced adenomas (advanced neoplasia were excluded to promote homogeneity). The Mann–Whitney *U* test compared the abundance—the peak area—of individual VOCs present in at least 50% of urine samples (to avoid possible over‐ or underestimation of the results). Discriminatory VOCs were classified against Pubchem and the Human Metabolome Database.

## 3 RESULTS

### 3.1 Patient characteristics

In total, 98 individuals with Lynch syndrome were included in the study and analysed with FAIMS and GC‐IMS, of whom 20 random individuals were also analysed with GC‐TOF‐MS (Figure 1, Tables 1, S1). Among inclusions, 58% were female, the median age was 51 years (IQR 40–61), 12% had a personal history of CRC, and 77% had undergone at least two previous colonoscopies, with a median surveillance interval of 26 months (IQR 24–30). Pathogenic germline variants in *MSH2*, *MSH6* and *PMS2* were each present in approximately 30% of individuals, while *MLH1* variants were present in approximately 10% of individuals.

**TABLE 1:** Patient characteristics, *n* (%) or median (interquartile range).

	To evaluate neoplasia detection			To evaluate follow‐up after polypectomy		
	Relevant neoplasia (*n* = 34)	Controls (*n* = 64)	*p*‐value	Relevant neoplasia (*n* = 24)	Controls (*n* = 43)	*p*‐value
Male	16 (47)	25 (39)	.445	13 (54)	18 (42)	.333
Age	58 years (47–64)	49 years (38–57)	.015	57 years (41–66)	50 years (38–59)	.141
Pathogenic variant						
*MLH1*	6 (18)	6 (9)	.667	4 (17)	5 (12)	.574
*MSH2*	10 (29)	20 (31)		7 (29)	15 (35)	
*MSH6*	11 (32)	18 (28)		9 (38)	11 (26)	
*PMS2*	7 (21)	19 (30)		4 (17)	12 (28)	
*EPCAM*	–	1 (2)		–	–	
History of colorectal cancer	6 (18)	6 (9)	.332	4 (17)	4 (9)	.443
History of bowel resection						
No	28 (82)	58 (91)	.457	20 (83)	39 (91)	.506
Left hemicolectomy	–	1 (2)		–	1 (2)	
Right hemicolectomy	5 (15)	4 (6)		3 (13)	3 (7)	
Proctectomy or sigmoidectomy	1 (3)	1 (2)		1 (4)	–	
Number of previous colonoscopies^a^						
0	6 (18)	6 (9)	.315	4 (17)	2 (5)	.267
1	2 (6)	8 (13)		1 (4)	4 (9)	
2+	25 (76)	50 (78)		19 (79)	37 (86)	
Surveillance interval	25 months (23–31)	26 months (24–30)	.985	25 months (20–29)	26 months (24–31)	.345
Comorbidity						
Diabetes mellitus type I or II	1 (3)	–	.347	1 (4)	–	.358
Hypertension	2 (6)	6 (9)	.710	1 (4)	4 (9)	.647
Medication use in the 3 months prior to sample collection						
Oral antibiotics	–	–	–	1 (4)	–	.358
Proton pump inhibitors	6 (18)	6 (9)	.332	6 (25)	3 (7)	.060
Laxatives	2 (6)	4 (6)	1.000	2 (8)	1 (2)	.290
Probiotics	4 (12)	2 (3)	.178	4 (17)	2 (5)	.177
Vitamin supplements	18 (53)	36 (56)	.754	9 (38)	23 (54)	.209
Body mass index						
18.5–25 kg/m^2^	17 (50)	46 (72)	.067	14 (58)	30 (70)	.571
25–30 kg/m^2^	10 (29)	13 (20)		6 (25)	9 (21)	
≥30 kg/m^2^	7 (21)	5 (8)		4 (17)	4 (9)	
Smoking status						
Smoker	6 (18)	7 (11)	.442	5 (21)	4 (9)	.329
Ex‐smoker (not smoked for >6 months)	9 (27)	24 (38)		6 (25)	16 (37)	
Never smoked	19 (56)	33 (52)		13 (54)	23 (54)	
Diet						
Regular diet	31 (91)	54 (84)	.663	21 (88)	35 (81)	.830
Vegetarian	3 (9)	8 (13)		3 (13)	7 (16)	
Other^b^	–	2 (3)		–	1 (2)	
Urine collection season						
Winter	11 (32)	19 (30)	.597	8 (33)	10 (23)	.769
Spring	9 (27)	19 (30)		6 (25)	11 (26)	
Summer	3 (9)	11 (17)		6 (25)	11 (26)	
Autumn	11 (32)	15 (23)		4 (17)	11 (26)	
Number of relevant neoplasia at study colonoscopy per patient						
1	17	n.a.	n.a.	13	n.a.	n.a.
2	13			8		
3+	4			3		
Most relevant neoplasia at study colonoscopy						
Colorectal cancer	2	n.a.	n.a.	n.a.	n.a.	n.a.
Advanced adenoma	3			3		
Advanced serrated lesion	1			0		
Non‐advanced adenoma	28			21		

^a^ Cumulative percentage is not 100% due to some missing values.

^b^ Other dietary habits included vegan, gluten‐free and/or lactose‐free.

Among the 98 individuals, 34 (35%) had relevant neoplasia at colonoscopy. The most relevant neoplasia was CRC in 2/98 (2.0%; one proximal and one distal pT1N0M0 adenocarcinoma), advanced adenoma in 3/98 (3.1%; all ≥10 mm with low‐grade dysplasia), advanced serrated lesion in 1/98 (1.0%; sessile serrated lesion ≥10 mm without dysplasia), and non‐advanced adenoma in 28/98 (28.6%, Table S2). The precancerous neoplasia were located in the proximal colon in 44% and had mostly sessile (59%) or flat (28%) morphology. Individuals with relevant neoplasia were older than controls (*p*‐value .015) but other characteristics did not significantly differ (Table 1).

### 3.2 Diagnostic accuracy

**FIGURE 2:**
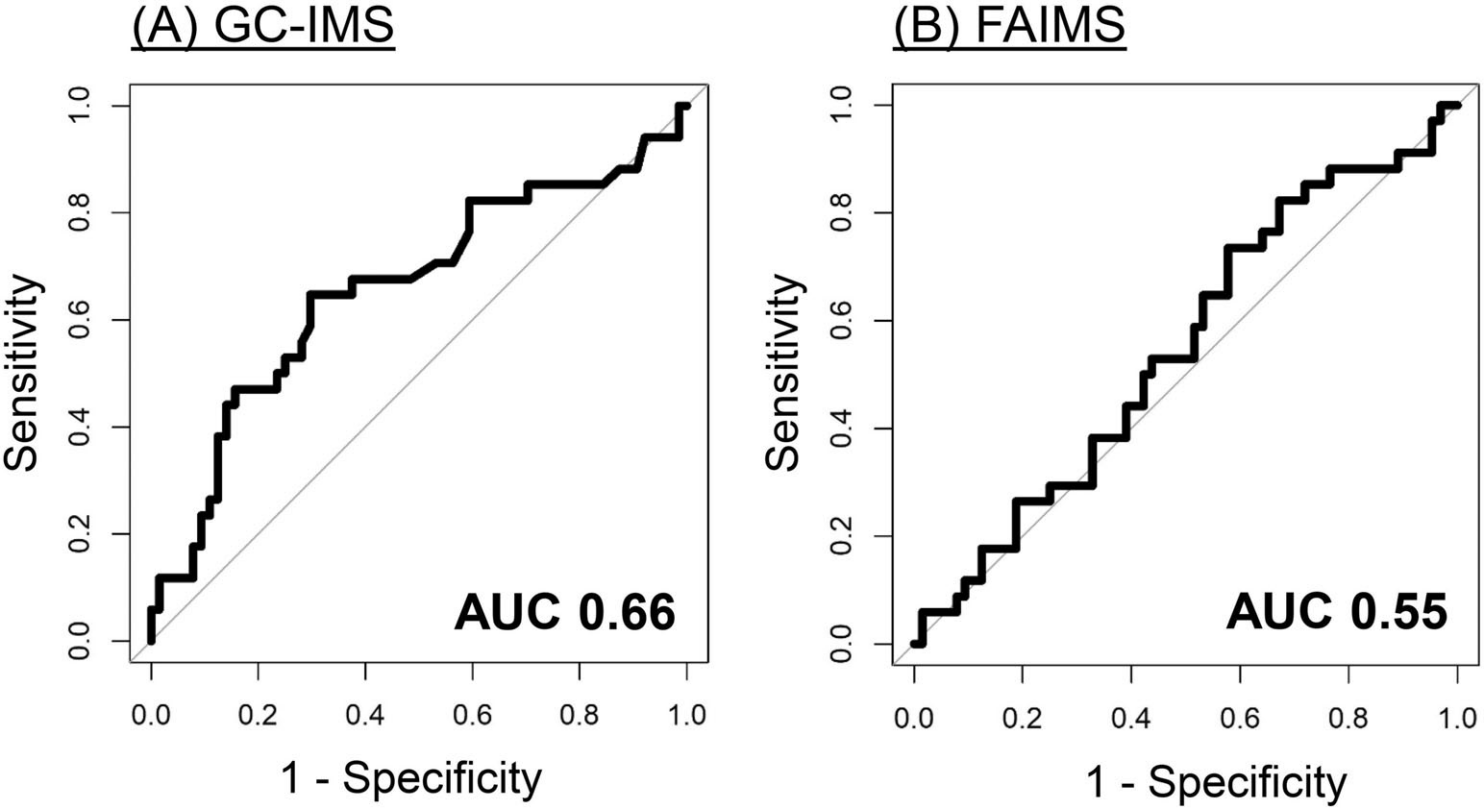
Receiver operating characteristic curves including area under the curve (AUC) to detect relevant colorectal neoplasia in Lynch syndrome by urinary volatile organic compounds, as analysed with (A) gas chromatography‐ion mobility spectrometry (GC‐IMS), and (B) field asymmetric ion mobility spectrometry (FAIMS).

Respective sensitivity and negative predictive value (NPV) to detect relevant colorectal neoplasia by urinary VOC patterns were 65% and 79% for GC‐IMS, and 74% and 75% for FAIMS, while specificity was 70% for GC‐IMS and only 42% for FAIMS (Table 2, Figure 2). Sensitivity increased and specificity decreased when correcting for gender, age, smoking habits, and dietary habits on sensitivity analysis (Table 2, Figure S1). We could not assess diagnostic accuracy for advanced neoplasia due to insufficient sample numbers.

**TABLE 2:** The accuracy of urinary volatile organic compounds, as analysed with GC‐IMS or FAIMS, to detect relevant colorectal neoplasia in Lynch syndrome.

	Machine learning algorithm^a^	Sensitivity (95% CI)	Specificity (95% CI)	Negative predictive value (95% CI)	Positive predictive value (95% CI)	Area under the curve (95% CI)
GC‐IMS						
Entire cohort	XGBoost	65% (46–80)	70% (58–81)	79% (66–88)	54% (38–69)	0.66 (0.54–0.78)
Sensitivity analysis^b^	Random forest	91% (75–98)	44% (26–62)	82% (56–95)	62% (46–75)	0.64 (0.50–0.78)
FAIMS						
Entire cohort	Sparse logistic regression	74% (56–87)	42% (30–55)	75% (57–87)	40% (28–54)	0.55 (0.43–0.67)
Sensitivity analysis^b^	Gaussian process	97% (84–100)	28% (14–47)	90% (54–99)	57% (43–70)	0.58 (0.44–0.73)

Abbreviations: CI, confidence interval; FAIMS, field asymmetric ion mobility spectrometry; GC‐IMS, gas chromatography‐ion mobility spectrometry.

^a^ Results are demonstrated for the best performing machine learning algorithm, out of the five algorithms tested (sparse logistic regression, Gaussian process, XGBoost, support vector machine, random forest).

^b^ In the sensitivity analyses, the neoplasia prevalence did not reflect reality, so the negative and positive predictive values are meaningless.

### 3.3 Diagnostic accuracy compared to faecal VOCs

Next, we compared the diagnostic accuracy for relevant neoplasia between urinary and faecal VOC patterns analysed by GC‐IMS in a sub‐cohort of 50 individuals who had collected both urine and faeces, of whom 2 (4.0%) had CRC, 1 (2.0%) advanced adenoma, 0 advanced serrated lesion, and 17 (34%) non‐advanced adenoma (Table S3). Urinary VOC patterns showed lower diagnostic accuracy (70% sensitivity, 70% NPV, 47% specificity) than faecal VOC patterns (95% sensitivity, 95% NPV, 67% specificity). Of note, both specificities differed from those observed in the entire cohorts, showing improved specificity for urinary VOCs (Table 2) and decreased specificity for faeces VOCs (see our previous publication^15^) at comparable sensitivities.

### 3.4 Follow‐up after polypectomy

**FIGURE 3:**
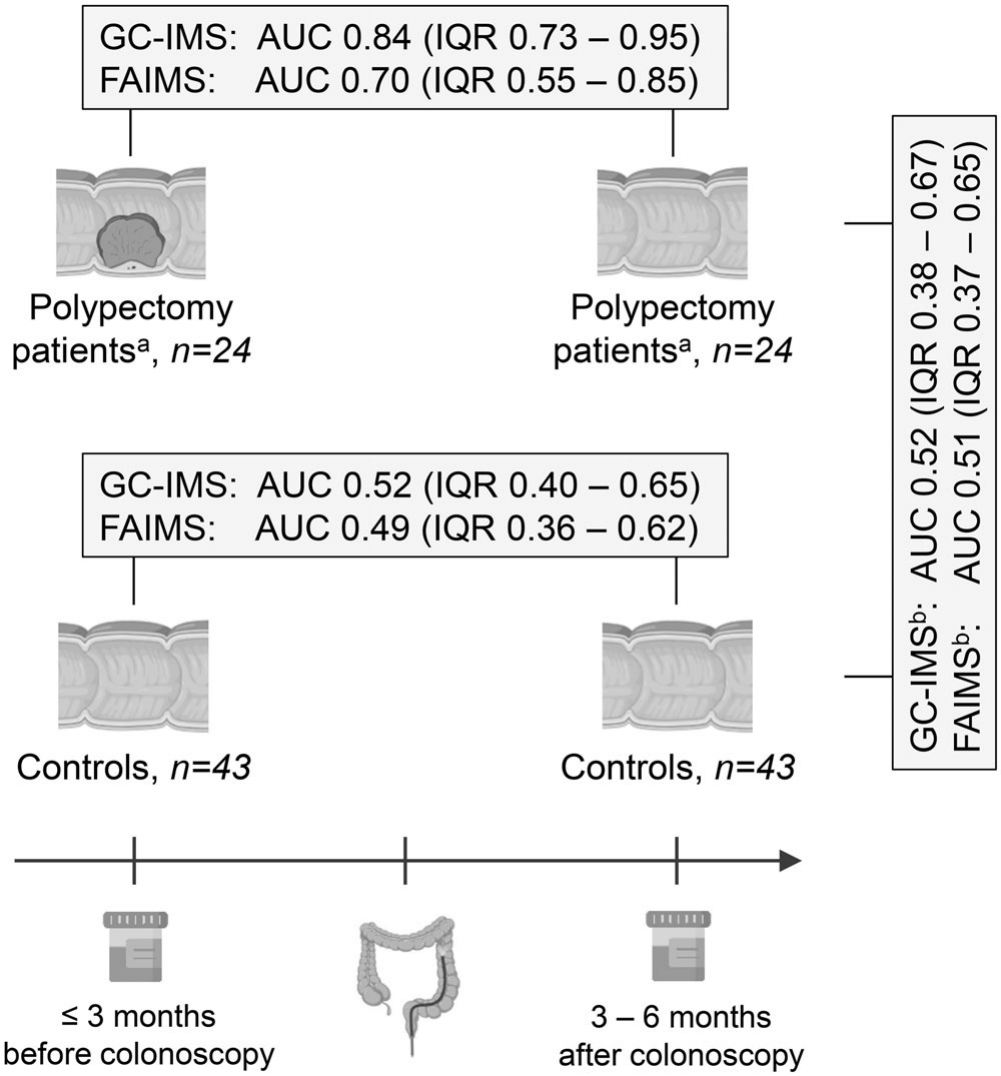
The performance of urinary volatile organic compounds, as analysed with GC‐IMS or FAIMS, for intra‐individual follow‐up after polypectomy in Lynch syndrome. Samples collected before and after normal colonoscopy were analysed (“controls”) as well as samples collected before and after complete removal of adenomas (“polypectomy patients”). Abbrevations: AUC, area under the curve; FAIMS, field asymmetric ion mobility spectrometry; GC‐IMS, gas chromatography‐ion mobility spectrometry; IQR, interquartile range. ^a^Of the 24 polypectomy patients, 3 (13%) had advanced adenomas and 21 (88%) non‐advanced adenomas—all were resected en‐bloc. ^b^We also compared samples collected after colonoscopy of 19 controls and 19 matched polypectomy patients, of which 3/19 (16%) had advanced adenomas and 16/19 (84%) non‐advanced adenomas, resulting in AUCs of 0.50 (IQR 0.31–0.69) for GC‐IMS and 0.61 (IQR 0.42–0.81) for FAIMS.

The performance of urinary VOC patterns for intra‐individual follow‐up after polypectomy was evaluated in 43 controls and 24 individuals with complete removal of advanced adenomas (*n* = 3) or non‐advanced adenomas (*n* = 21, Figure 3). We observed that VOC patterns differed before and after polypectomy—especially when analysed by GC‐IMS rather than by FAIMS (AUC 0.84 and 0.70, respectively)—while they were similar before and after normal colonoscopy (‘controls’, AUC 0.49–0.52). Following polypectomy, VOC patterns resembled those of ‘controls’ (AUC 0.51–0.52), which in the case of GC‐IMS was also observed when correcting for gender, age, smoking habits, and dietary habits (GC‐IMS: AUC 0.50, FAIMS: AUC 0.61).

It should be noted that the feature location plots showed that the VOCs discriminating before and after polypectomy were within the chemical output for FAIMS, but mostly related to the reactive ion peak for GC‐IMS, indicating that there was not sufficient distinguishing power of other true peaks (Figure S2).

### 3.5 Adenoma‐specific VOCs

Among the 20 samples of individuals with non‐advanced adenomas and controls, GC‐TOF‐MS identified 1513 unique individual VOCs, of which 1400 were excluded from further statistical analysis because they were present in <50% of samples. Upon statistical analysis of the remaining 113 VOCs, the abundance of decanoic acid—a medium‐chain fatty acid—was significantly increased in individuals with non‐advanced adenomas compared to controls (*p*‐value .023, Table S4).

## 4 DISCUSSION

This prospective longitudinal study evaluated the potential of urinary VOCs as non‐invasive patient‐friendly biomarkers to guide optimal colonoscopy intervals in Lynch syndrome, aiming to reduce colonoscopy burden and post‐colonoscopy CRC rates in this population. Diagnostic accuracy of urinary VOC patterns (“the VOC‐fingerprint”) for relevant colorectal neoplasia, comprising mostly non‐advanced adenomas, was poor for FAIMS and moderate for GC‐IMS, with the latter showing 65% sensitivity, 79% NPV and 70% specificity. Interestingly, VOC patterns differed before and after polypectomy, while after polypectomy VOC patterns resembled those of controls. Nevertheless, the diagnostic accuracy of urinary VOC patterns was influenced by sample size and external confounders, and was lower compared to faecal VOC patterns among individuals who had collected both urine and faeces.

Given the accelerated adenoma‐carcinoma sequence in Lynch syndrome, non‐invasive biomarkers should have high sensitivity to detect CRC, advanced adenomas, and ideally non‐advanced adenomas, accepting suboptimal specificity. Because our study population was already under strict colonoscopy surveillance, the diagnostic accuracy of VOCs was mostly based on non‐advanced adenomas ≤5 mm (82% of relevant neoplasia assessed). The diagnostic accuracy of urinary VOC patterns for sporadic adenomas has previously been assessed in two studies, although neither differentiated accuracy between non‐advanced and advanced adenomas. In these studies, GC‐IMS showed 58% sensitivity and 62% specificity to distinguish 80 adenomas (% non‐advanced adenomas unknown) from 37 controls within the national CRC screening programme,^34^ while FAIMS showed 91% sensitivity and 15% specificity in a large symptomatic cohort of 406 controls and 94 adenomas (71% non‐advanced adenomas).^35^ The lower accuracy of FAIMS compared to GC‐IMS in these studies, as well as in ours, may be attributable to differences in analytical technology and the chemical window analysed. With regard to CRC detection, urinary VOC patterns may be accurate biomarkers, given that our recent meta‐analysis showed pooled sensitivity and specificity of 84% (95% confidence interval 73%–91%) and 70% (95% confidence interval 63%–77%), respectively.^25^ This accuracy may be underestimated due to the use of older systems for VOC analysis and heterogeneity among included studies. To determine the potential of urinary VOC patterns for colorectal surveillance in Lynch syndrome, further studies with larger cohorts of individuals with Lynch syndrome are needed to validate our diagnostic accuracy for non‐advanced adenomas and to evaluate the diagnostic accuracy for advanced adenomas and CRC.

Sub‐analysis indicated that relevant neoplasia was detected with considerably lower sensitivity and specificity by urinary VOC patterns compared to faecal VOC patterns. If true, the lower accuracy would outweigh the advantages of urine over faeces in clinical practice, but further evaluation is required as this sub‐analysis may be biased by differences in storage duration and temperature between urine and faeces samples (median 11 months at −80°C versus median 13 months at −20°C, respectively). Remarkably, the specificities observed in this sub‐analysis differed from those observed in the entire cohorts. This may be explained by (minor) differences in patient characteristics and neoplasia prevalence, which possibly have a greater impact on outcomes of machine learning algorithms when sample size decreases. In this regard, we observed in the current study and our previous study^15^ that the accuracy of urinary and faecal VOC patterns either increased or decreased when correcting for patient characteristics (i.e., gender, age, smoking and diet). The above‐mentioned findings underline the need for further studies investigating which confounders—including patient characteristics and sampling and analytical variables—predominantly affect the accuracy of VOC patterns and how these confounders can be corrected for. Next, international experts should develop an evidence‐based protocol for sample preparation, sample analysis and data analysis, for example using the Delphi Method, to ensure accuracy, head‐to‐head comparison and reproducibility of further studies necessary to advance VOCs towards clinical application.

In the current exploratory study, urinary VOC patterns—especially when analysed by GC‐IMS—normalised after successful polypectomy. These outcomes provide evidence that adenoma presence may result in distinct urinary VOC patterns and that urinary VOC patterns may be useful biomarkers for intra‐individual follow‐up after polypectomy, aiming to early detect missed or incompletely resected adenomas and thereby potentially decrease post‐colonoscopy CRC in Lynch syndrome. Yet, the promising outcomes of GC‐IMS were mostly based on VOCs related to the reactive ion peak, which in currently used GC‐IMS systems is susceptible to sample and instrumental variations hampering clinical application. Nevertheless, this may have been the result of the relatively small sample size, as it was not observed in similar studies of our group on faecal VOCs including moderately sized cohorts of individuals at average risk or with Lynch syndrome.^15^, ^36^ To determine the potential of urinary VOC patterns for intra‐individual follow‐up after polypectomy in Lynch syndrome, our findings should be validated by other research groups in larger cohorts of individuals with Lynch syndrome.

In addition to the potential of VOC patterns, we explored whether VOC identification on a molecular level by GC‐TOF‐MS could be useful for non‐invasive detection of colorectal neoplasia in Lynch syndrome and for gaining insights into the pathomechanism of these lesions. We found that the presence of non‐advanced adenomas was associated with increased urinary abundance of decanoic acid, which is a medium‐chain fatty acid. Although this finding was based on a small number of adenomas, increased abundance of decanoic acid has also been previously observed in faeces and blood of individuals with CRC or advanced adenomas, with AUCs to detect CRC ranging between 0.67 and 0.82.^37^, ^38^, ^39^ Decanoic acid seems to display antineoplastic activity by upregulating genes involved in apoptosis while downregulating genes involved in proliferation.^40^ Besides decanoic acid, multiple other urinary VOCs have shown significantly different abundance in the presence of colorectal neoplasia, yet none have shown consistent results across studies, probably reflecting methodological and geographical variability.^25^

Acknowledged limitations of our study are its single centre design and lack of sample size calculation because previous data on urinary VOCs for adenoma detection were scarce and conflicting. Due to its exploratory nature, this study included a moderately sized cohort of 98 individuals with Lynch syndrome, leading to rather wide confidence intervals for diagnostic accuracy, independent of analytical system or correction for external confounders. Another limitation is that we were unable to investigate whether it is important to correct urinary VOC analysis for sample water content (for example by creatinine level) or for batch effects (we analysed samples in three batches to analyse them within 12 months considering stability concerns^30^). A strength of this study is that it is the first study evaluating the diagnostic potential of urinary biomarkers for colorectal neoplasia in Lynch syndrome, using a prospective longitudinal design wherein urine was collected in a standardised manner before and after high‐quality colonoscopy. Methodology was consistent with key considerations for VOC analysis^30^, ^41^, ^42^ and involved collection of midstream spot urine after overnight fasting to reduce the influence of water content, vaginal microbiota, and confounders like diet and smoking. Lastly, we evaluated the accuracy of various advanced systems for VOC analysis and the robustness of our results through sensitivity analyses.

To conclude, this exploratory study shows that urinary VOC patterns hold promise as non‐invasive biomarkers for postponing colonoscopy and for intra‐individual follow‐up after polypectomy in Lynch syndrome, in an effort to reduce post‐colonoscopy CRC rates and colonoscopy burden. To determine the clinical suitability of urinary VOC patterns as a patient‐friendly alternative to faecal biomarkers for colorectal neoplasia detection, there is a need for large validation studies by different research groups that also assess stability and accuracy compared to faecal VOC patterns. Next to urinary VOC patterns, our study suggests that VOC identification on a molecular level may be useful for non‐invasive detection of colorectal neoplasia in Lynch syndrome and for gaining insights into the pathomechanism of these lesions.

## AUTHOR CONTRIBUTIONS

**Elsa L. S. A. van Liere:** Conceptualization; writing – original draft; methodology; project administration; formal analysis. **Dewkoemar Ramsoekh:** Conceptualization; writing – review and editing; funding acquisition; methodology; supervision. **Trenton K. Stewart:** Methodology; writing – review and editing; formal analysis. **Emma Daulton:** Writing – review and editing; methodology; formal analysis. **Maarten A. J. M. Jacobs:** Conceptualization; methodology; writing – review and editing; supervision. **Evelien Dekker:** Conceptualization; writing – review and editing; methodology; supervision. **Sofie Bosch:** Conceptualization; writing – review and editing; methodology. **James A. Covington:** Conceptualization; writing – review and editing; methodology; formal analysis; supervision. **Tim G. J. de Meij:** Conceptualization; methodology; writing – review and editing. **Nanne K. H. de Boer:** Conceptualization; funding acquisition; writing – review and editing; methodology; supervision.

## FUNDING INFORMATION

This study was funded by Vaillant Fonds and Dutch Digestive Foundation (MLDS, grant number WO 19‐05). The funders of the study had no role in study design, data collection, data analysis, data interpretation, or writing of the report.

## CONFLICT OF INTEREST STATEMENT

ELSAvL, TKS, Emma D, MAJMJ, SB and JAC declare no competing interests. DR has received a research grant (unrestricted) from AbbVie, outside the submitted work. He has served as a member of the data safety monitoring board of the VIVIAD trial. Evelien D has endoscopic equipment on loan from FujiFilm and has received a research grant from FujiFilm outside the submitted work. She has received honoraria for consultancy from FujiFilm, Olympus, InterVenn, Ambu, Norgine and Exact Sciences, and speakers' fees from Olympus, GI Supply, Norgine, IPSEN, Steris and FujiFilm. She is chair of the CRC Screening Committee of World Endoscopy Organisation and the Dutch Post‐Polypectomy Surveillance Guideline, Member Post‐Polypectomy Surveillance Guideline of the European Society of Gastrointestinal Endoscopy. TGJdM has served as a speaker for Nutricia, Mead Johnson and Winclove. He has served as an advisory board member for Nutricia. NKHdB has served as a speaker for AbbVie and MSD and has served as a consultant and principal investigator for TEVA Pharma BV and Takeda. He has received a research grant (unrestricted) from Dr. Falk, TEVA Pharma BV, Dutch Digestive Foundation (MLDS) and Takeda; all outside the submitted work.

## ETHICS STATEMENT

The study was approved by the Research Ethical Committee of Amsterdam UMC (2020.317). All study participants provided written informed consent. The study was registered at the WHO International Clinical Trials Registry Platform (NL8749).

## Supporting information


**APPENDIX S1:** Supporting information.

## Data Availability

All source code is publicly available on GitHub (https://github.com/Warwick-Sensors-Lab/R-Code-Warwick-Analyser). Other data that support the findings of this study can be made available from the corresponding author upon reasonable request.
