# Supplementary material for: The diagnostic potential of urinary volatile organic compounds for colorectal neoplasia in Lynch syndrome—A prospective longitudinal study
Source: Int J Cancer. 2025 Sep 23;158(1):257–66. doi: 10.1002/ijc.70140 (PMC12588553; doi:10.1002/ijc.70140)
Supplement: Supplementary file 1 — APPENDIX S1: Supporting information. [file IJC-158-257-s001.pdf]

THE DIAGNOSTIC POTENTIAL OF URINARY VOLATILE ORGANIC COMPOUNDS FOR COLORECTAL NEOPLASIA IN LYNCH SYNDROME – A PROSPECTIVE LONGITUDINAL STUDY

AUTHORS

Elsa L.S.A. van Liere; Dewkoemar Ramsoekh; Trenton Stewart; Emma Daulton; Maarten A.J.M. Jacobs; Evelien Dekker; Sofie Bosch; James A. Covington; Tim G.J. de Meij; Nanne K.H. de Boer

TABLE OF CONTENTS

Analytical methodology of each system for VOC-analysis	page 2
Table S1: Characteristics of patients and adenomas analysed with GC-TOF-MS	page 3
Table S2: Characteristics of colorectal neoplasia analysed with GC-IMS and FAIMS	page 5
Figure S1: ROC curves of the sensitivity analyses	page 6
Table S3: Difference in diagnostic accuracy for relevant colorectal neoplasia between urinary and faecal VOCs	page 7
Figure S2: Feature location plots of VOCs discriminating individuals before and after polypectomy	page 8
Table S4: Individual urinary VOCs analysed by GC-TOF-MS discriminating non-advanced adenomas from controls	page 9
References	page 10

ANALYTICAL METHODOLOGY OF EACH SYSTEM FOR VOC-ANALYSIS

GC-IMS analysis

The GC-IMS system used in our study was a FlavourSpec™ (G.A.S., Germany), which has an integrated autosampler (CTC Analytics, Switzerland). Total runtime per sample was 15 minutes. The system's analytical methodology was similar to that in our previous study and is described in detail in the supplementary file of our previous study[1].

GC-TOF-MS analysis

The GC-TOF-MS used in our study consists of a TRACE 1300 GC (Thermo Fisher Scientific, United Kingdom) and BenchTOF-HD (Markes International, United Kingdom). These are coupled to a Centri thermal desorption unit and an autosampler (Markes International, United Kingdom). Total runtime per sample was 25 minutes. The system's analytical methodology was similar to that in our previous study and is described in detail in the supplementary file of our previous study[1].

FAIMS analysis

The FAIMS system used in our study was The Lonestar™ (Owlstone, United Kingdom), fitted with an ATLAS system to elevate the sample temperature and with a Split Flow Box System to set the flow rate across the sample and the make-up air. The system's analytical methodology aligns with that of previous similar studies conducted by our group[2,3].

Prior to analysis, each sample was thawed at room temperature for 30 minutes and then heated at 40°C for 10 minutes. Upon entering the Lonestar, VOCs are first ionised by a Nickel-63 radiation source after which they are transported using a carrier gas (clean compressed air at around 0.2 MPa) between two plates to which an asymmetric electric field is applied. A compensation voltage is applied to prevent that VOCs impact a plate and as such lose their charge and are not detected.

The temperature settings were as follows: 35°C for sample, 70°C for lid, and 120°C for filter. The flow rate over the sample was 0.5L/min which was mixed with 1.5L/min additional clean air, resulting in a 2L/min total flow rate. The dispersion field intensity was swept between 0% and 100% in 51 steps and the compensation voltage between +6 V and −6 V in 512 steps. The resulting output consisted of 52,224 data points per run. Using the "fast scan" set-up, each sample was run ten times sequentially, of which the second run was used for data analysis. In between urine samples, a blank sample was analysed 25 times sequentially to prevent carry over between urine samples. Total runtime for one urine sample and one blank sample was 8 minutes.

Table S1. Characteristics of patients and non-advanced adenomas analysed with gas chromatography time-of-flight mass spectrometry, *n* (%) or median (IQR). Differences between groups were not assessed statistically because of the low number of cases in each group.

	**Adenomas** (n=10)	**Controls** (n=10)
**Male**	5 (50)	1 (10)
**Age**	60 years (50 – 64)	55 years (40 – 65)
**Pathogenic variant**		
MLH1	3 (30)	-
MSH2	2 (20)	3 (30)
MSH6	2 (20)	5 (50)
PMS2	3 (30)	2 (20)
**History of colorectal cancer**	2 (20%)	2 (20%)
**History of bowel resection**		
No	7 (70)	8 (80)
Left hemicolectomy	-	1 (10)
Right hemicolectomy	2 (20)	1 (10)
Proctectomy or sigmoidectomy	1 (10)	-
**Number of previous colonoscopies**		
0	1 (10)	1 (10)
1	-	2 (20)
2+	9 (90)	7 (70)
**Surveillance interval**	25 months (16 – 30)	29 months (24 – 33)
**Comorbidity**		
Diabetes Mellitus type I or II	-	-
Hypertension	-	2 (20)
**Medication use in the 3 months prior to sample collection**		
Oral antibiotics	-	-
Proton pump inhibitors	3 (30)	1 (10)
Laxatives	1 (10)	1 (10)
Probiotics	1 (10)	1 (10)
Vitamin supplements	5 (50)	6 (60)
**Body mass index**		
18.5 – 25 kg/m^2	6 (60)	7 (70)
25 – 30 kg/m^2	2 (20)	2 (20)
≥ 30 kg/m^2	2 (20)	1 (10)
**Smoking status**		
Smoker	2 (20)	1 (10)
Ex-smoker *(not smoked for >6 months)*	4 (40)	3 (30)
Never smoked	4 (40)	6 (60)
**Diet**		
Regular diet	8 (80)	9 (90)
Vegetarian	2 (20)	1 (10)
**Urine collection season**		
Winter	3 (30)	4 (40)
Spring	-	1 (10)
Summer	-	4 (40)
Autumn	7 (70)	1 (10)
**Number of adenomas at study colonoscopy per patient**		
1	7	n.a.
2	2	
3	1	
**Location adenoma**		
Caecum	2	n.a.
Ascending, incl. hepatic flexure	2	
Transverse	2	

Descending, incl. splenic flexure<br>Sigmoid	2<br>2	
**Paris classification for morphology**<br>Sessile (Is)<br>Flat or flat elevated (IIa or IIb)	 8<br>2	 n.a.
**Size adenoma**	3 mm (2 – 3)	n.a.

Table S2. Characteristics of relevant precancerous colorectal neoplasia analysed with gas chromatography – ion mobility spectrometry and field asymmetric ion mobility spectrometry, *n* or median (IQR).

	**TO EVALUATE NEOPLASIA DETECTION**			**TO EVALUATE FOLLOW-UP AFTER POLYPECTOMY**	
	**Advanced adenomas** (n=3)	**Non-advanced adenomas** (n=28)	**Advanced serrated lesions** (n=1)	**Advanced adenomas** (n=3)	**Non-advanced adenomas** (n=21)
**Location**					
Caecum	-	3	-	-	3
Ascending, incl. hepatic flexure	-	5	-	-	4
Transverse	-	5	1	-	3
Descending, incl. splenic flexure	1	7	-	1	5
Sigmoid	1	8	-	1	6
Rectum	1	-	-	1	-
**Size**	12 mm (10 – 15)	3 mm (2 – 4)	10 mm	12 mm (10 – 15)	3 mm (2 – 4)
**Paris classification for morphology**					
Pedunculated (Ip)	1	2	-	1	1
Sessile (Is)	1	17	1	1	13
Flat or flat elevated (IIa or IIb)	1	8	-	1	7
Missing	-	1	-	-	-
**High-grade dysplasia**	-	n.a.	n.a.	-	n.a.
**Villous component**	1	n.a.	n.a.	1	n.a.
**Type of serrated lesion**					
Sessile serrated lesion without dysplasia	n.a.	n.a.	1	n.a.	n.a.

Figure S1. Receiver operating characteristic curves to detect relevant colorectal neoplasia in Lynch syndrome by urinary volatile organic compounds on sensitivity analysis. *GC-IMS = gas chromatography – ion mobility spectrometry, FAIMS = field asymmetric ion mobility spectrometry.*

GC-IMS

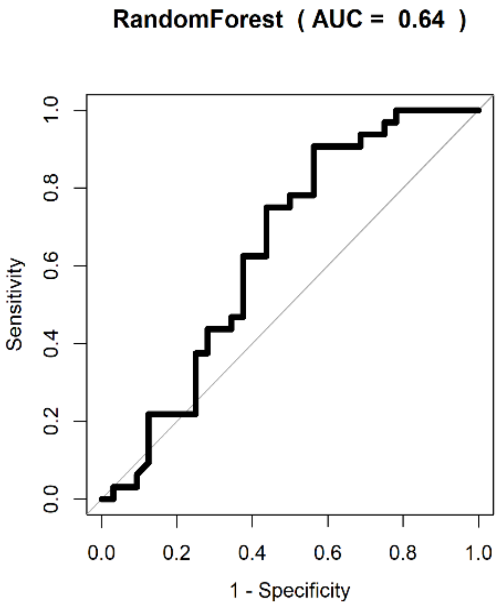

FAIMS

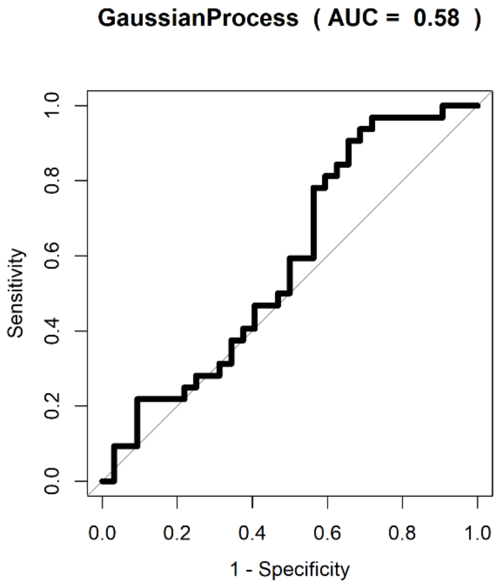

Table S3. The difference in diagnostic accuracy for relevant colorectal neoplasia[a] in Lynch syndrome between urinary and faecal volatile organic compounds (VOCs) as analysed with gas chromatography – ion mobility spectrometry.

	**Machine learning algorithm** [b]	**Sensitivity** (95%-CI)	**Specificity** (95%-CI)	**Negative predictive value** (95%-CI)	**Positive predictive value** (95%-CI)	**AUC** (95%-CI)
Urinary VOCs	Sparse logistic regression	70% (46 – 88)	47% (28 – 66)	70% (46 – 87)	47% (29 – 65)	0.51 (0.34 – 0.68)
Faecal VOCs	Gaussian process	95% (75 – 100)	67% (47 – 83)	95% (74 – 100)	66% (46 – 81)	0.84 (0.72 – 0.96)

AUC = area under the curve.

a. Among the 50 individuals who had collected both urine and faeces, 20 (40%) had relevant neoplasia at study colonoscopy. The most relevant neoplasia was CRC in 2/50 (4.0%), advanced adenoma in 1/50 (2.0%), advanced serrated lesion in 0/50 and non-advanced adenoma in 17/50 (34%).

b. Results are demonstrated for the best performing machine learning algorithm, out of the five algorithms tested (sparse logistic regression, Gaussian process, XGBoost, support vector machine, random forest).

Figure S2. Feature location plots of the VOCs discriminating individuals before and after polypectomy. *FAIMS = field asymmetric ion mobility spectrometry, GC-IMS = gas chromatography – ion mobility spectrometry.*

<u>FAIMS</u>

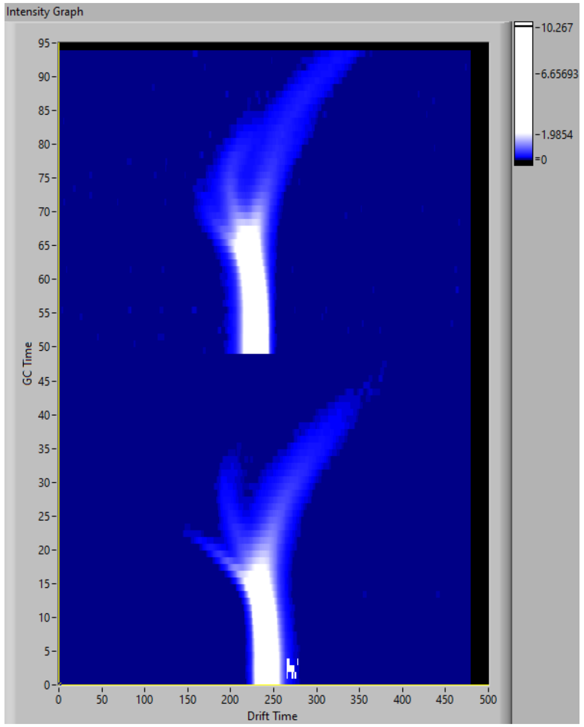

<u>GC-IMS</u>

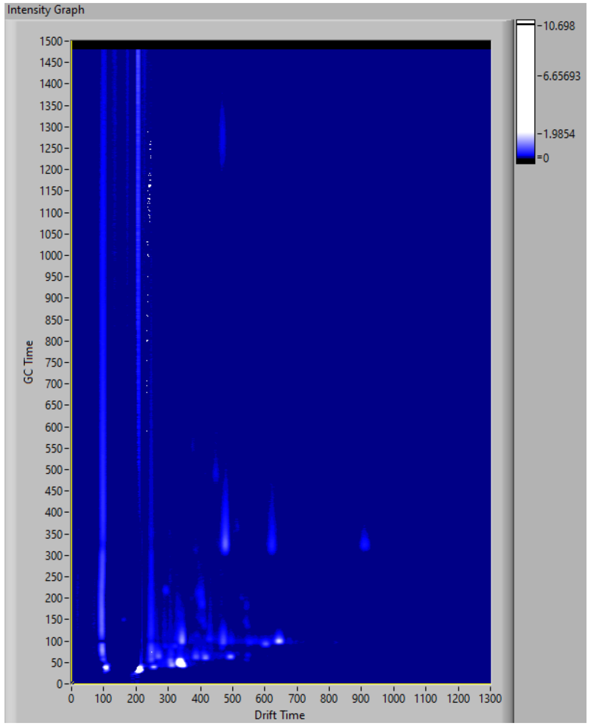

Table S4. Individual urinary volatile organic compounds, as analysed with gas chromatography time-of-flight mass spectrometry, that differed significantly between individuals with non-advanced adenomas and controls.

	**Volatile organic compound**[a] *(standardised names)*	**Chemical class**	**Retention time**, *min.*	**Increased/ decreased in adenomas**	**Abundance – adenomas**, *median counts (IQR)*	**Abundance – controls**, *median counts (IQR)*	**p-value**
Human	Decanoic acid	Medium-chain fatty acids	9.5341	Increased	8.1×10^5 (4.7×10^5 – 2.1×10^6)	3.6×10^5 (1.3×10^5 – 6.2×10^5)	0.023
Non-human	Tris(2-chloropropyl) phosphate	Trialkyl phosphates	12.5002	Increased	2.7×10^5 (2.4×10^5 – 4.1×10^5)	1.2×10^5 (9.5×10^4 – 2.4×10^5)	0.019

a. To avoid over- or underestimation of the results, only VOCs that were present in ≥50% of the samples analysed were included for further statistical analysis. This criterion resulted in a selection of 113 different VOCs. Of note, upon statistical analysis of all 1513 individual urinary VOCs identified, no additional VOCs were discriminatory between individuals with non-advanced adenomas and controls.

REFERENCES

1. van Liere ELSA, Ramsoekh D, Daulton E, et al. Faecal Volatile Organic Compounds to Detect Colorectal Neoplasia in Lynch Syndrome-A Prospective Longitudinal Multicentre Study. *Aliment Pharmacol Ther* 2024.
2. Arasaradnam RP, McFarlane MJ, Ryan-Fisher C, et al. Detection of colorectal cancer (CRC) by urinary volatile organic compound analysis. *PLoS One* 2014; **9**(9): e108750.
3. Mozdiak E, Wicaksono AN, Covington JA, Arasaradnam RP. Colorectal cancer and adenoma screening using urinary volatile organic compound (VOC) detection: early results from a single-centre bowel screening population (UK BCSP). *Tech Coloproctol* 2019; **23**(4): 343-51.
